# ALK-fusion-positive non-small cell lung cancer concomitant with 5q deletion myelodysplastic syndrome: a case report

**DOI:** 10.3389/fonc.2026.1909434

**Published:** 2026-08-19

**Authors:** Mohammad Hassan Hodroj, Hiba Moukadem

**Affiliations:** 1 Department of Medical Oncology, Cedars Cancer Centre, McGill University Health Center, Montreal, QC, Canada; 2 Division of Hematology and Oncology, Department of Internal Medicine, American University of Beirut Medical Center, Beirut, Lebanon

**Keywords:** ALK-fusion, del 5q, MDS, MPMN, NSCLC

## Abstract

Multiple primary malignant neoplasms (MPMNs) refers to the concurrent or sequential development of at least two distinct primary malignant tumors within the body, arising from the same organ or different organs. The current manuscript discusses the unique clinical case of a female patient diagnosed with concomitant metastatic ALK-positive NSCLC and del 5q-MDS and was treated for both entities with oral systemic treatments only with a remarkable response for both diseases.

## Introduction

Lung cancer is currently the leading cause of cancer deaths worldwide, with non-small cell lung cancer (NSCLC) representing approximately 85% of all lung cancer cases. Only 2% to 7% of NSCLC cases are caused by the oncogenic gene rearrangements of anaplastic lymphoma kinase (*ALK*), labeled as *ALK*-positive NSCLC (1). Patients with the *ALK*-positive subtype are commonly diagnosed at an advanced stage, with brain metastasis being at the top of the metastatic sites list, and *ALK* inhibitors being the oral standard of care therapy (2).

In contrast, myelodysplastic syndromes (MDSs) are myeloid neoplasms characterized by morphologically abnormal and ineffective maturing hematopoiesis, leading to peripheral blood cytopenia that can affect one, two, or all three hematopoietic lineages and carrying the risk of progression into acute leukemia (3). MDS with the deletion of the long arm of chromosome 5 (*del 5q*) is the only MDS subtype defined by a cytogenetic alteration, constituting 10% to 20% of all MDS cases, and is characterized by anemia that is commonly severe and macrocytic. Lenalidomide, an oral immunomodulator, is the selective standard of care treatment for this specific subtype of MDS (4).

Multiple primary malignant neoplasms (MPMNs) refer to the concurrent or sequential development of at least two distinct primary malignant tumors within the body, arising from the same organ or different organs. It is classified as simultaneous MPMNs, if they occur with an interval of ≤6 months, or metachronous MPMNs, if the interval is >6 months (5). In the current study, we report a case of a female patient diagnosed with concomitant metastatic *ALK*-positive NSCLC and *del 5q* MDS. A written informed consent was obtained from the patient before proceeding with the report.

**Figure 1:**
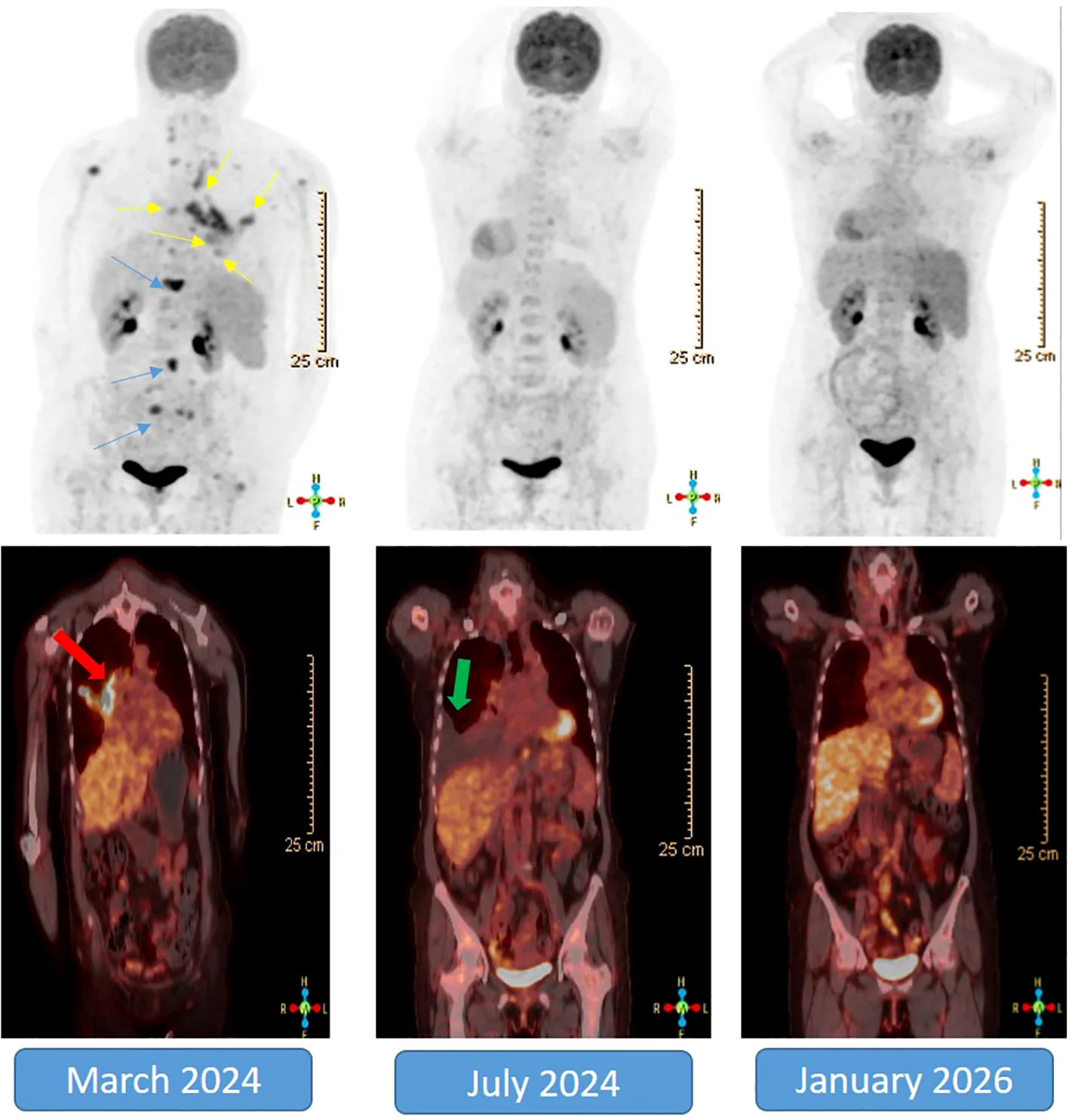
Response to treatment on FDG-PET scan. The red arrow points to the primary FDG-avid malignant lesion in the right middle lobe, with the avidity resolving by July 2024 and remaining resolved in January 2026. The green arrow points to the right pleural effusion present at diagnosis, which had resolved by the January 2026 scan. Blue arrows point to the lytic bone lesions seen at diagnosis that had resolved on follow-up scans. Yellow arrows point to the FDG-avid hilar and mediastinal lymph nodes seen at diagnosis that had resolved on follow-up scans.

**Figure 2:**
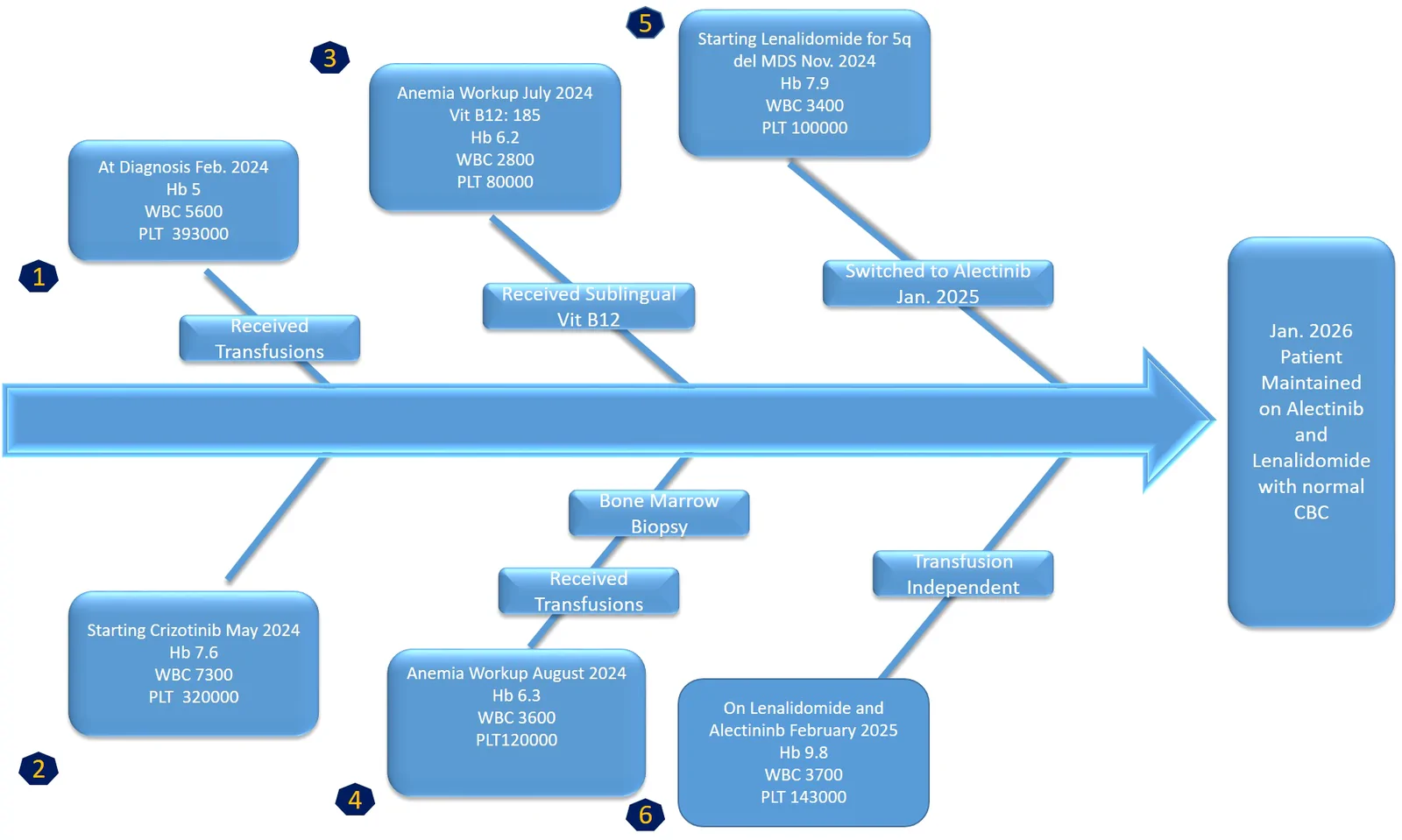
Chronological timeline of the diagnosis and clinical treatment of both ALK-positive NSCLC and 5q del MDS, with corresponding complete blood count results. CBC, complete blood count; Hb, hemoglobin in g/dL; WBC, white blood count in cells/μL; PLT, platelets in platelets/μL; Vit B12, vitamin B12 in pg/mL, and 5q del MDS, 5q deletion myelodysplastic syndromes.

A 62-year-old female patient who has never smoked, known to have hypertension, type-2 diabetes mellitus, dyslipidemia, and a history of pericarditis, presented in January 2024 to the pulmonary outpatient department at the American University of Beirut Medical Center (AUBMC) with worsening shortness of breath, normocytic anemia, severe fatigue, and generalized bone pain. Chest X-ray showed a large right-sided pleural effusion that was aspirated and revealed adenocarcinoma cells on cytology. She was referred to the oncology outpatient department in February 2024 with a chest CT scan showing a central hilar and suprahilar right peribronchovascular soft-tissue infiltrate continuing into a mass measuring 6 cm × 2.4 cm, causing narrowing of the bronchi, with recurrent right pleural effusion and mediastinal lymphadenopathy. The patient developed worsening anemia with her Hb level of 5 g/dL requiring transfusion of three units of packed red blood cells. The patient had severe financial limitations, which delayed several investigations. Fluorodeoxyglucose positron emission tomography (FDG-PET) scan was performed in March 2024, showing a right middle lobe avid mass of 3.6 cm × 2.1 cm, maximum standardized uptake value (SUVmax) of 12.3, compatible with primary malignancy, large right pleural effusion, several mediastinal lymphadenopathies, and extensive lytic metastatic bone lesions in the axial and appendicular skeleton. A needle biopsy of the primary lung mass demonstrated invasive, poorly differentiated adenocarcinoma with thyroid transcription factor-1 (TTF-1) positive, p40 negative, programmed death-ligand 1 (PD-L1) of 20%, and ALK (D5F3) strongly and diffusely positive. Given her diffuse metastatic disease and the *ALK*-positive status, brain magnetic resonance imaging (MRI) was performed and was negative for metastasis. The patient was started in May 2024 on crizotinib 250 mg orally twice daily, since it was the only available ALK inhibitor due to the economic crisis in Lebanon. A few weeks after treatment initiation, some of her symptoms started to improve, but she continued to require pleural taps and blood transfusion. A repeat PET scan in July 2024 revealed a remarkable response to treatment, and the patient also improved clinically. Initially, her anemia was correlated to the extensive metastatic disease of the bone involving the bone marrow. However, she continued to be transfusion-dependent despite the significant improvement in the osseous lesions. Her anemia was investigated, showing a vitamin B12 deficiency at a level of 185 pg/mL, for which she received adequate replacement. However, the patient was still transfusion-dependent every 2 weeks, with a hemoglobin (Hb) level of 6.2 g/dL, and she developed leukopenia, with a white blood cell (WBC) count of 2,800 cells/μL, together with thrombocytopenia, with a platelet count of 80,000/μL, prompting a bone marrow biopsy. A bone marrow biopsy was performed in September 2024, demonstrating a 95% cellular marrow with trilineage dysplasia, erythroid hypoplasia, and 2%–3% blasts, consistent with low-blast MDS. Cytogenetic analysis by karyotype showed del 5q, 46,XX,*del(5)(q12q33)*[20]. The biopsy was negative for metastatic carcinoma. Her Revised International Prognostic Scoring System (IPSS-R) score was 4 (intermediate risk), for which she was started on lenalidomide 10 mg orally once daily for 21 days of a 28-day cycle in November 2024. A repeat PET scan in December 2024 showed resolution of the hilar mass, stable right pleural effusion, and treated metastatic bone lesions. **Figure 1** demonstrates the treatment response on FDG-PET scan. Two months after initiating lenalidomide, the patient’s transfusion frequency decreased to once every 4 weeks, and then she became transfusion-independent in February 2025 with her Hb level reaching 10.5 g/dL, and platelet count improving to 143,000/μL. Once the standard-of-care alectinib became available in Lebanon, she was switched to alectinib 600 mg orally twice daily in January 2025, and she has maintained a good response to treatment. Currently, she remains on alectinib and lenalidomide. Her last PET scan in January 2026 revealed stable disease, and her complete blood count was normal, with an Hb of 13.1 g/dL, a WBC count of 6,200 cells/μL, and a platelet count of 216,000/μL. **Figure 2** summarizes the timeline for the patient’s diagnosis, clinical treatment, and associated complete blood count results.

## Discussion

Reviewing the literature, there are very limited data on concurrent lung cancer and MDS, with most cases being delayed and secondary MDS following chemotherapy and radiation therapy. However, few cases of MDS occurring concurrently with solid tumors have been reported. Victoria et al. reported a case of a 76-year-old male ex-smoker with limited-stage small cell lung cancer and concurrent MDS with trisomy 8, who was managed with azacitidine (6). Another case reported a 66-year-old male smoker with metastatic epidermal growth factor receptor (*EGFR; exon 19*)-mutated NSCLC and secondary MDS identified on next-generation sequencing, which showed chromosome 7 and *5q deletions*. The patient was treated with azacitidine for anemia and thrombocytopenia (7). Unlike those two cases, our patient is a woman with no history of smoking, but she shared the same age range (>60 years). Similarly, she had multilineage cytopenia. However, she was treated with lenalidomide, the approved oral targeted therapy for lower-risk MDS based on the results of the MDS-003 phase II clinical trial, which showed the effectiveness of lenalidomide in reducing transfusion requirements (8). As in the case reported by Victoria et al., our patient presented with concurrent symptoms of both lung cancer and MDS, highlighting the possibility of simultaneous MPMNs. However, in our patient, the official diagnosis of MDS was confirmed by biopsy 7 months after the diagnosis of NSCLC due to financial constraints, thus, classifying the case as metachronous MPMN.

The novelty of our case lies in its rarity and the combination of both *ALK* fusion-positive NSCLC and *del 5q* MDS, both of which can be treated by oral targeted therapies at home without the need for hospital admissions or infusions. Moreover, starting the treatment with a first-generation ALK inhibitor as crizotinib, achieving an excellent response, and then switching to alectinib after 8 months, was necessitated by drug availability and the patient’s financial situation. This approach did not align with the standard of care in 2024, which was lorlatinib, as supported by the phase III CROWN trial or alectinib based on the ALEX phase III trial (2, 9). Reviewing the literature, there were no data or documented evidence to date, about a direct potential physiological, genetic, or biological link between the co-occurrence of *ALK*-positive NSCLC and *del 5q* MDS. However, this does not exclude a possible linkage that remains undetectable by current diagnostic assays, is not well established yet, or has not been reported. Fluorescence *in situ* hybridization (FISH) and genetic sequencing were not performed because of the patient’s financial situation, despite their potential diagnostic benefits. The challenge for our patient remains the duration of response that she might maintain for both entities. Additional genetic sequencing may be highly beneficial for our patient to identify other possible genetic alterations that may increase the risk of developing additional malignancies.

## Conclusion

To our knowledge, this is the first case report to describe the concurrent occurrence of *ALK-*positive NSCLC and *del 5q* MDS, both successfully treated with oral targeted therapies. Our case alerts medical oncologists and hematologists to the importance of assessing MPMNs where clinically indicated. MPMNs do not include solid tumors exclusively, but may also include hematologic malignancies. Clinicians should consider extensive hematologic assessment in patients with persistent unusual anemia or cytopenias that do not align with the response to treatment, basic hematologic workup, or known side effects. It also highlights the feasibility of treating two different malignancies simultaneously with oral therapies, without the need for hospital admission; when adequate diagnostic studies are performed, an accurate diagnosis is reached, and the patient is treated using a multidisciplinary approach. Avoiding admissions in appropriate circumstances decreases the economic burden on patients and the healthcare system without affecting the patient’s treatment course. The case of our patient was limited by financial constraints, which delayed needed investigations and precluded genetic sequencing. Genetic sequencing, when available, is essential for identifying underlying oncogenic genetic alterations in cancer patients and may help diagnose or guide the management of some tumors. Furthermore, the case underscores the importance of investigating new or suspicious symptoms in patients first rather than automatically correlating or attributing them to a known malignancy.

## Data Availability

The original contributions presented in the study are included in the article/Supplementary Material. Further inquiries can be directed to the corresponding author.
